# Grinder Injury of the Hand: A Rare but Devastating Occupational Hazard

**DOI:** 10.1055/s-0041-1735902

**Published:** 2021-10-22

**Authors:** Tushar Patial, Rajinder K. Mittal, Ramneesh Garg, Sheerin Shah, Amandeep Kaur

**Affiliations:** 1 Department of Plastic and Reconstructive Surgery, Dayanand Medical College and Hospital, Ludhiana, Punjab, India

**Keywords:** amputation, meat grinder injury, hand injuries, hand deformities

## Abstract

Food handlers and workers are exposed to several occupational hazards not frequented by the general population. Grinder injuries of the hand present a devastating consequence of industrial food processing that is infrequently described. Herein, we describe two cases that presented to our department with meat grinder injuries of the hand.

India exports ∼3,722 million dollars worth of meat every year. 1 Increasing income and availability of meat products have led to an increase in demand, consequent to which meat processing plants have witnessed a growth in the country. Since a large number of workers are employed in the industry, especially the unorganized sector, machine injuries are expected to see a continuous rise. We report two cases that presented to us with meat grinder injuries and their management.

## Case Report

### Case 1

**Fig. 1:**
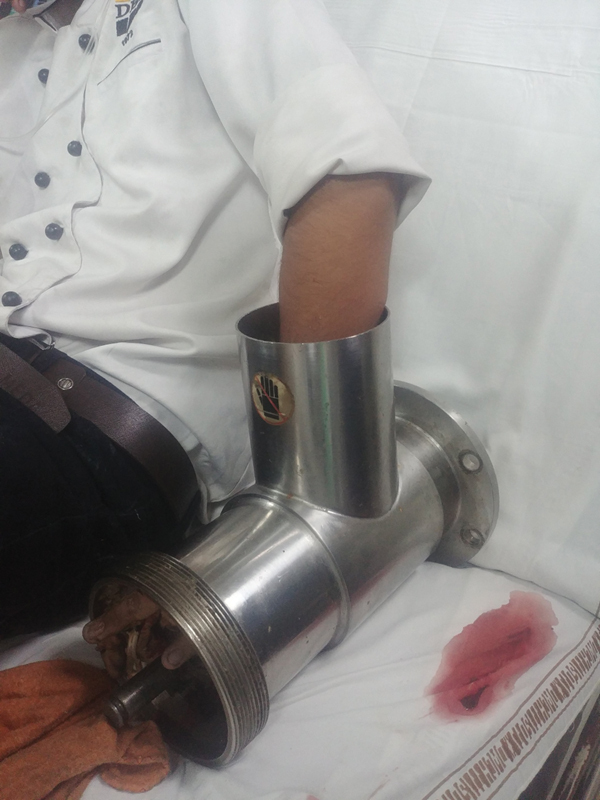
The left hand caught within the meat grinder.

**Fig. 2:**
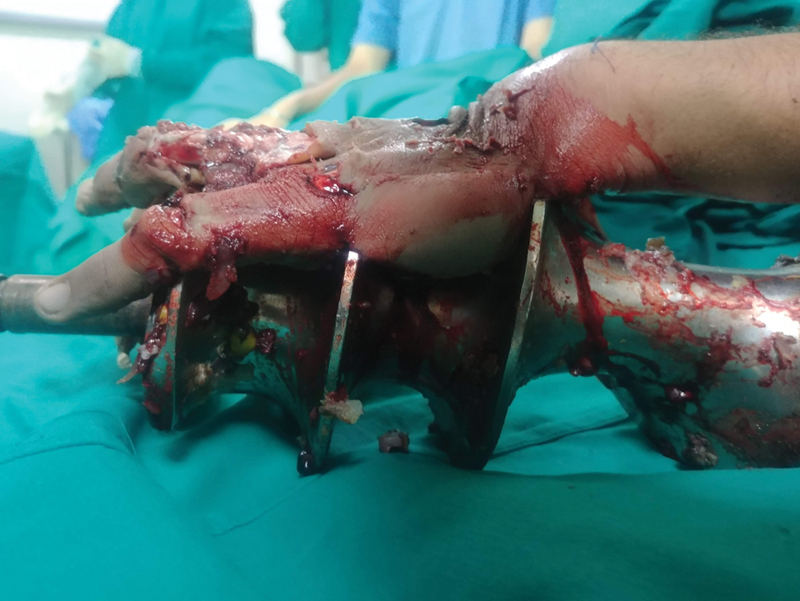
Lateral view of the left hand after removal of the outer shell.

**Fig. 3:**
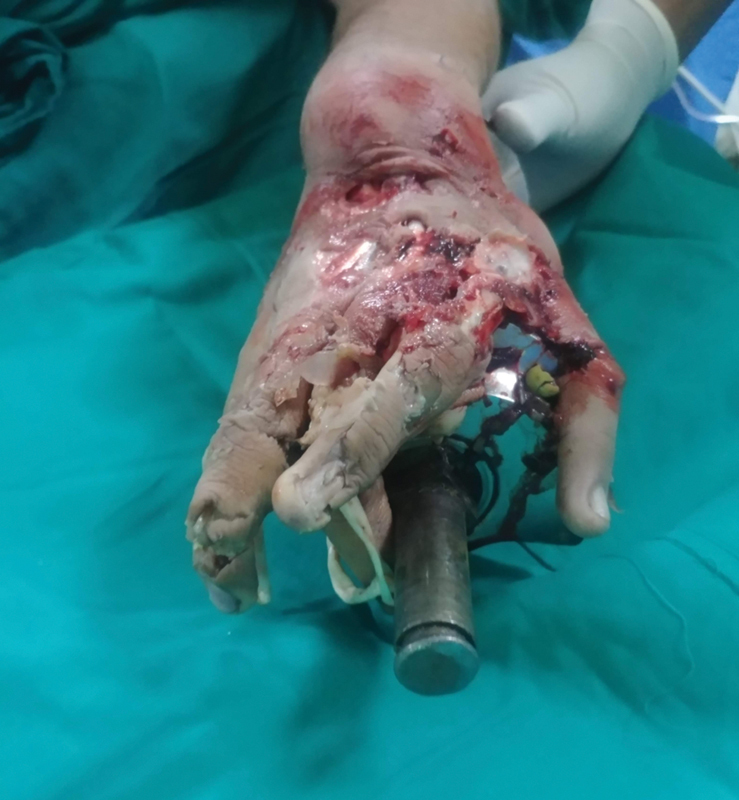
Dorsal view of the left hand after removal of the outer shell.

**Fig. 4:**
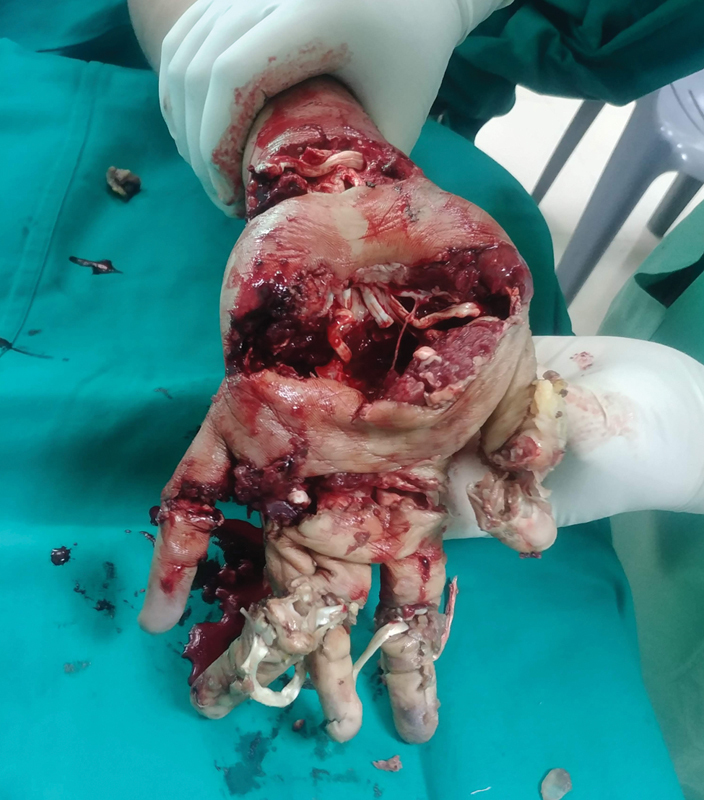
Palmar view of the left hand after removal of the outer shell.

**Fig. 5:**
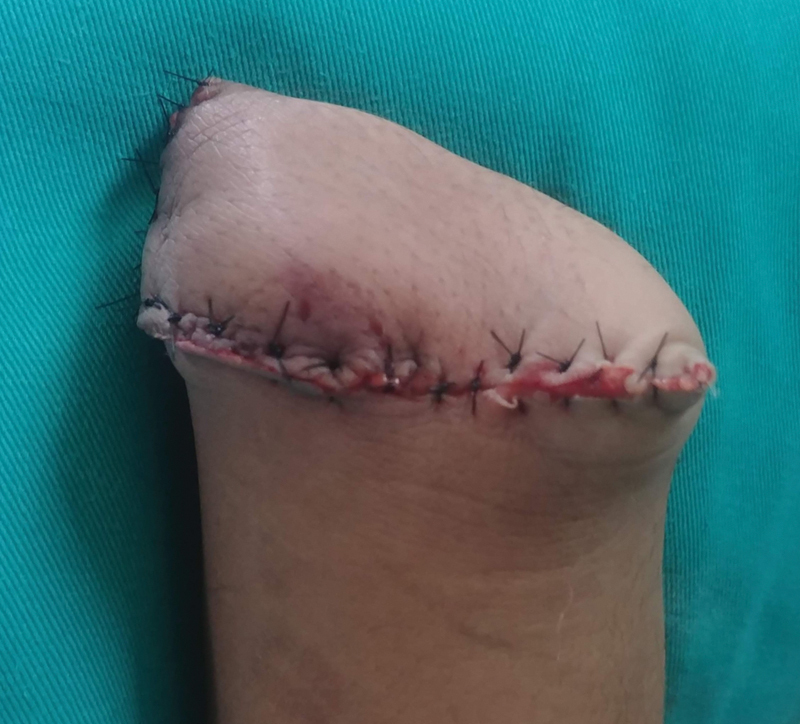
Postdisarticulation volar view.

A 22-year-old male patient presented to the emergency with his left hand caught in a commercial meat grinder. After partly dismantling the grinder, the patient was brought to our hospital, with the hand still caught in the grinder ( Fig. 1 ). The patient was taken to the operation theater and after giving general anesthesia, a welder was brought in to cut across the thick metallic components of the grinder using a cutting torch. After removal of the outer shell, the hand was found to have extensive crush injuries. Three discrete longitudinal wounds were present over the fingers, the distal palmar crease, and the distal wrist crease corresponding to the worm of the grinder ( Figs. 2 and 3 ). No bleeding was seen from the wounds till the level of the wrist and the bones of the distal to the wrist were completely crushed with exposure of the underlying tendons ( Fig. 4 ). After washing out the wound with povidone-iodine and saline, a disarticulation was performed at the level of the wrist ( Fig. 5 ). The postoperative period was uneventful, and the patient was discharged after 1 day. He was followed closely in the outpatient department but refused prosthesis and reconstructive surgery for the injury.

### Case 2

**Fig. 6:**
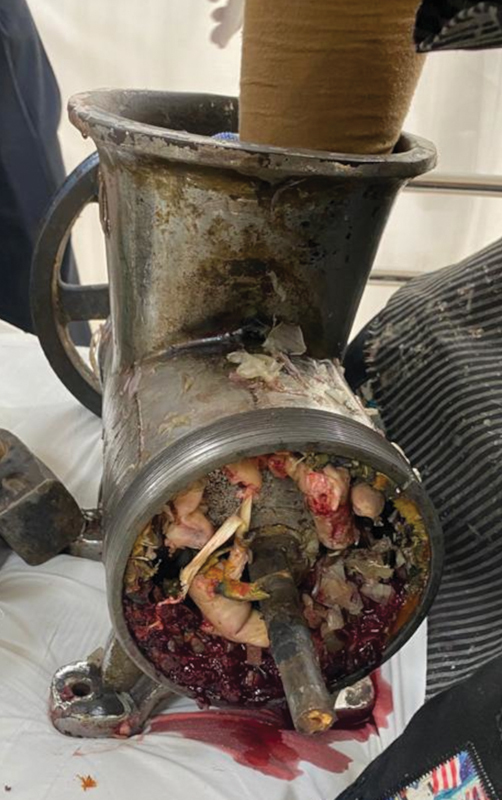
The right hand caught within the meat grinder, with visible crushing of hand.

**Fig. 7:**
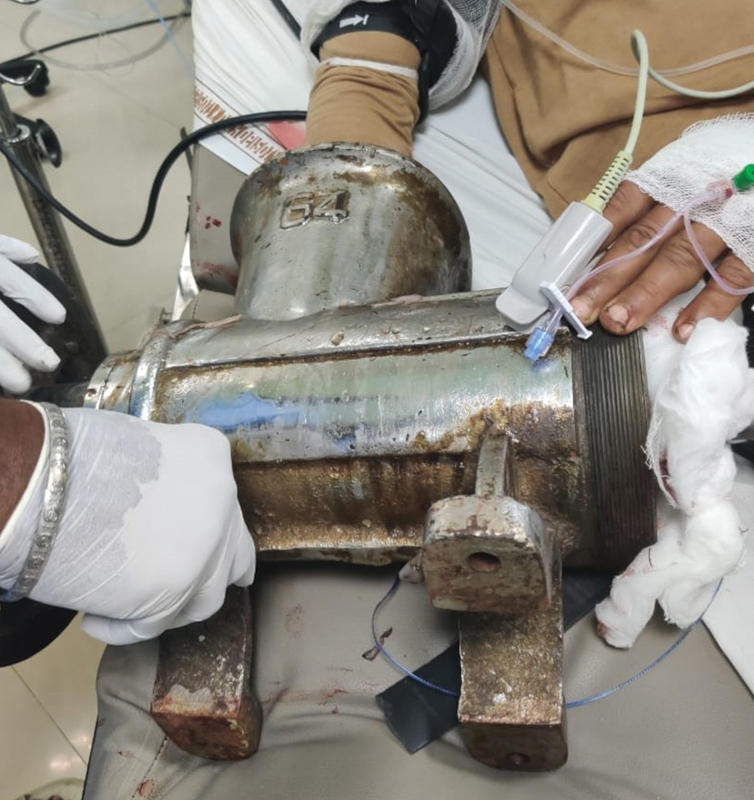
The right hand caught within meat grinder.

A 29-year-old male patient reported to the emergency with his right hand caught in a meat grinder machine. The grinder was still attached to the hand when the patient was brought in ( Figs. 6 and 7 ). The patient was taken to the operation theater and after anesthesia, the surgical team was able to extract the hand from the grinder by derotation of the worm of the grinder. Assessment of the hand revealed severe crushing of the small bones of the hands and nonreconstructible neurovasculotendinous injuries of the hand. After debridement and washing the wound, a disarticulation was performed at the level of the distal radioulnar joint.

After an uneventful postoperative period, suture removal was done at 14 days and currently the patient wears a cosmetic prosthesis.

## Discussion

Hands are an essential component of our existence. They are not only important for physical and psychological development but also play an essential role in our appearance and our professional career. 2 It is no surprising that injuries to the hands can have devastating consequences for an individual. Loss of just the thumb is equivalent to a 40% loss of hand function and a 25% loss of the whole body. 3

In the food industry, tasks that were traditionally performed by manual labor are being rapidly replaced by machines. When properly used, these machines can be very efficient at increasing productivity, although they can also have the effect of increasing the risk of accidents at the workplace, especially for those who are inadequately trained in their proper use. 4

Trauma is the most common cause of upper limb amputation. This puts younger people at risk of these injuries, not only due to extensive psychosocial burden but also due to severe functional impairments. 2 Reports of meat grinder injuries are uncommon in literature. In one series, by Brandner et al, all patients were under 40 years of age and the dominant hand was involved in all cases as in our patients. 5 In a similar series by Yildiran et al, 70% of all meat grinder injuries were seen in the pediatric age group. The authors suggested that this was perhaps due to the inquisitive nature of children. 6

Although sparse, literature on the topic reveals that in many cases, the patient is brought to the hospital with his/her hand firmly wedged in the meat grinder. 5 7 8 It is prudent to note that careful removal of the involved area from the grinder is crucial to limit further damage to the involved hand. 5 This is sometimes possible by reverse turning of the grinder as in the second case in our series. 7 9

After extraction of the hand from the machine, the goal should be to preserve the function of the hand as much as possible. With the advent of modern microsurgical techniques, the repair of neurovascular injuries of the hands is possible. However, in severely mutilated injuries of the hand, amputation is usually the rule. 5 For upper limb amputees, a prosthetic replacement has been the standard of care. This can be done as early as 3 months after the amputation, once the swelling has subsided. Of late, hand transplantation is increasingly being done at numerous centers around the world. However, owing to the high cost, availability of donors, prolonged rehabilitation, and the need for immunosuppression, this is seldom done. 2

To mitigate the morbidity associated with an injured limb, there is a need to educate the food handlers about the dangers of the machine. The cutting edges are often exposed and may cause serious injuries. However, there are measures that can be taken to reduce the danger, such as the use of feeding devices when operating the grinder. 4 Due to the nature of the machine, employers must ensure that the grinders are retrofitted with a primary safeguard. The workers should be trained to use a proper plunger when feeding meat into the grinder and to turn off and unplug the grinder when not in use. 4 10

All employees involved in the handling of the meat grinder should receive the job training under supervision, till they can work safely on their own. 10

## Conclusion

Meat grinder injuries lead to devastating consequences for the involved individual. Increased demand for meat will almost certainly lead to an increase in the incidence of such injuries. The employer must ensure that protective equipment is used. Sufficient supervised training and properly functioning machinery are essential in preventing such injuries. With the increase in need and supply of processed food, grinder injuries are likely to increase in incidence. The treatment for these injuries should aim to preserve as much of the limb as possible. Modern prosthesis can be extremely useful for those with an amputated upper limb; however, the patients should be referred to specialized centers where microsurgical facilities exist for reconstruction.
